# mTOR: A possible therapeutic target against SARS-CoV-2 infection

**Published:** 2021

**Authors:** Nabab Khan

**Affiliations:** Department of Biomedical Sciences, University of North Dakota, School of Medicine and Health Sciences, Grand Forks, North Dakota 58203, USA

The recent pandemic of SARS-CoV-2 has emerged as a health emergency to develop effective therapeutic strategies for restricting deadly disease, COVID-19. SARS-CoV-2 infects cells by the endocytosis process *via* receptor-mediated binding and priming by cellular proteases [1,2]. However, the virus replicates in autophagosomes like structures in the cytosol by escaping endolysosomes pathway and develops acute respiratory syndrome by inducing cytokine storms [3–6]. Endolysosomes are acidic organelles that contain ∼60 acid hydrolases capable of catalyzing the degradation of viral particles, enhancing endolysosome acidification might suppress SARS-CoV-2 infection [4,7–9]. The acidic nature of endolysosomes regulates endolysosomes’ functions and the autophagy degradation pathway [8,10,11]. Multiple endolysosomes-associated proteins such as v-ATPase (vacuolar-ATPase) [12,13], TRPML1 (mucolipin-1) and BK channels (Maxi-potassium) [14], two-pore channels [15], SLC38A9 (solute carrier family 38 member 9) [16–18], and mTOR (mammalian target of rapamycin) [19–21], regulate the acidic nature of lysosomes.

mTOR downstream signaling pathways regulate fundamental cellular processes such as metabolism, transcription, protein synthesis, apoptosis, cell cycle, endolysosomes, autophagy, and immune regulation and tolerance [22–26]. However, disturbed mTOR signaling is involved in various pathological conditions such as cardiovascular, cancer, inflammation, and metabolic disorders [23,26,27]. Besides, various viruses like influenza [28], HIV-1 [29,30], and coronaviruses, MERS-CoV [31] and SARS-CoV-2 [32–34], to complete viruses’ replication and life cycles, can hijack it. Recently, it has been identified that the SARS-CoV-2 virus exploits the mTOR-signaling pathway to progress the infection [33,35]; however, mTOR inhibitors suppress virus infection at a significant level with nanomolar concentrations [35]. The mTOR-signaling pathway has also been used to block several other viruses’ infection and replications by inducing autophagy and inhibiting viral protein synthesis [36–40]. Hence, mTOR could be an excellent therapeutic target to suppress the SARS-CoV-2 infection and COVID-19 using synthetic and natural compounds [41–43] (Figure 1). Thereby, various drugs are suggested and used to treat SARS-CoV-2 infection and COVID-19 pathogenesis like sapanisertib, metformin [44,45], rapamycin [46,47], and rapalog (everolimus) [39,48], which target the mTOR-signaling pathway. Recently, rapalog has been shown a protective role in small samples of COVID-19 aged patients [47–50]. Rapalog, an analog of rapamycin, is commonly used as an immunosuppressant [51]. However, it exerts immunostimulatory effects, for example, enhancing T-cell response in microbes’ infection and behaving as an immunoadjuvant in vaccination [52]. Hence, a placebo study should be conducted to explore and screen existed rapamycin like synthetic and natural compounds against SARS-CoV-2 infection and COVID-19 *in vitro* and *in vivo* conditions [43]. Table 1 contains the list of clinical trials of natural and synthetic mTOR inhibitors against COVID-19.

Here briefly concludes that the mTOR sensor might be a potential therapeutic target to suppress SARS-CoV-2 infection and its pathogenesis, COVID-19. Hence, mTOR inhibitors, synthetic and mainly naturally available compounds, should be screened to determine their potency to suppress SARS-CoV-2 infection and COVID-19.

## Figures and Tables

**Figure 1:**
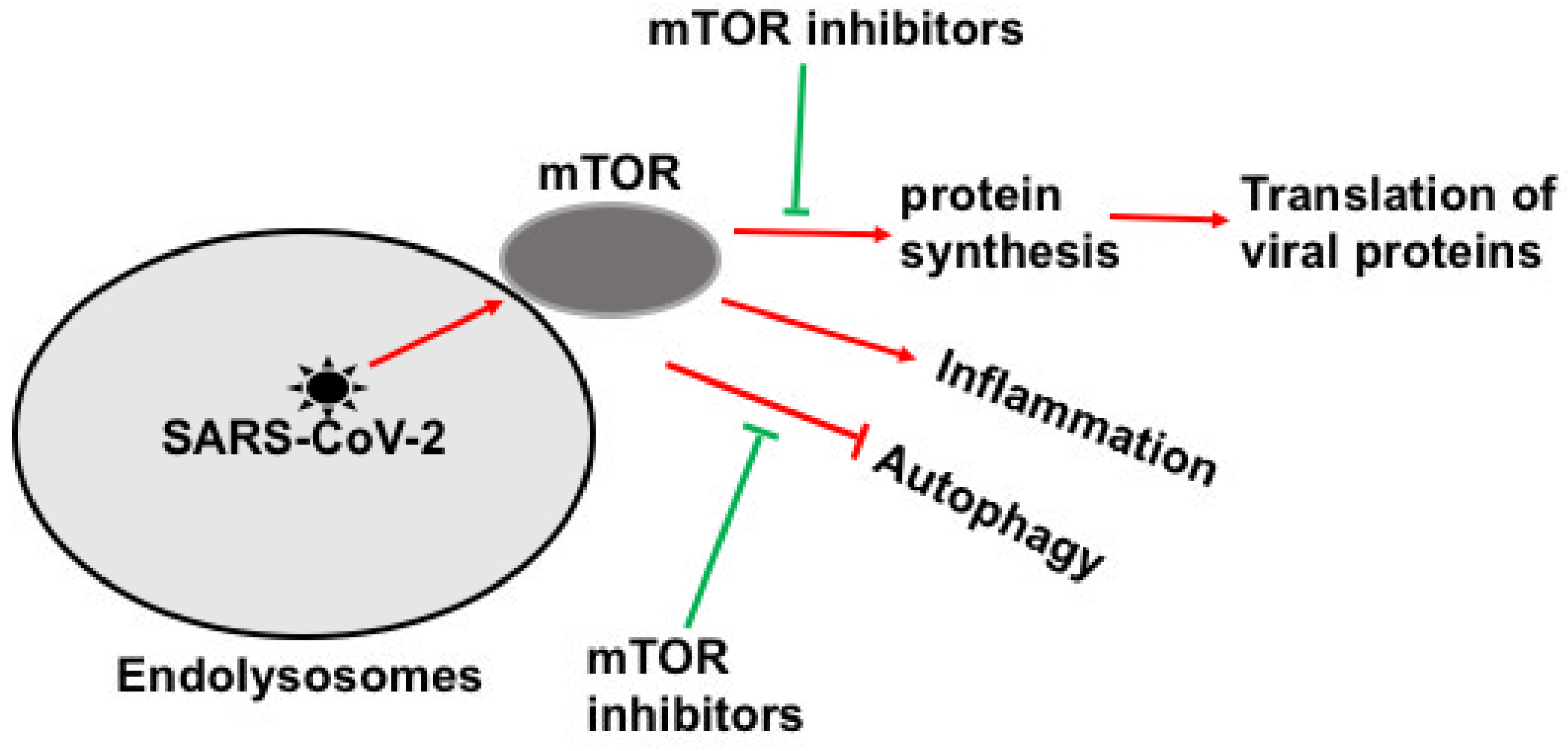
The mTOR sensor is exploited by SARS-CoV-2 for replication and survives in cells. However, mTOR inhibitors could suppress SARS-CoV-2 replication and COVID-19 by inducing autophagy, restricting the synthesis of viral proteins and inflammation.

**Table 1:** Clinical trials of natural and synthetic mTOR inhibitors against COVID-19.

mTOR inhibitors	Clinical trials’ References
Sirolimus	NCT04461340
Sirolimus	NCT04341675
Sirolimus	NCT04371640
RTB101	NCT04409327
Metformin	NCT04604678
Resveratrol [43]	NCT04542993
Quercetin [43]	NCT04377789
